# Nonischemic Cardiac Manifestations in VEXAS Syndrome

**DOI:** 10.1001/jamanetworkopen.2024.50251

**Published:** 2024-12-12

**Authors:** Marie Robert, Alexis Mathian, Valentin Lacombe, Hervé Lobbes, Léopold Adélaïde, Maelle Le Besnerais, Jean-Nicolas Dacher, Benjamin Terrier, Arsène Mékinian, Rim Bourguiba, Sophie Georgin-Lavialle

**Affiliations:** 1 Department of Internal Medicine, Université Paris-Cité, Hôpital Bichat-Claude Bernard, Assistance Publique-Hôpitaux Paris, Paris, France; 2 Assistance Publique-Hôpitaux Paris, Hôpital Pitié-Salpêtrière, Institut E3M, Service de Médecine Interne 2, Centre de Référence des Maladies Auto-Immunes et Auto-Inflammatoires Systémiques Rares de L’adulte d’Ile-de-France, Centre et Martinique, INSERM, Centre d’Immunologie et des Maladies infectieuses-Paris (CIMI-Paris), Paris, France; 3 Department of Internal Medicine and Clinical Immunology, Centre Hospitalier Universitaire Angers, Angers, France; 4 Department of Internal Medicine, Centre Hospitalier Universitaire Estaing, Clermont-Ferrand, France; 5 Department of Internal Medicine, Centre Hospitalier de Vienne, Vienne, France; 6 Department of Internal Medicine, Centre Hospitalier Universitaire Charles Nicolle, Rouen, France; 7 Department of Radiology, Université de Normandie, UNIROUEN, INSERM 1096, Centre Hospitalier Universitaire Rouen, Rouen, France; 8 Department of Internal Medicine, Université Paris-Cité, Hôpital Cochin, Assistance Publique-Hôpitaux Paris, Paris, France; 9 Department of Internal Medicine, Sorbonne Université, Hôpital Saint-Antoine, Assistance Publique-Hôpitaux Paris, Paris, France; 10 Hôpital des Forces de Sécurité de l’Intérieur, La Marsa, Université Tunis el Manar, Faculté de Médecine de Tunis, Tunis, Tunisie; 11 Department of Internal Medicine, CEREMAIA, Sorbonne Université, Hôpital Tenon, Assistance Publique-Hôpitaux Paris, Paris, France

## Abstract

This case series reports on the clinical presentation, laboratory findings, imaging characteristics, treatments, and outcomes of nonischemic cardiac manifestations in patients with VEXAS syndrome with confirmed pathogenic *UBA1* alterations.

## Introduction

VEXAS (vacuoles, E1 enzyme, X-linked, autoinflammatory, somatic) syndrome is associated with somatic variations in the *UBA1* gene within hematopoietic progenitor cells.^1^ It is characterized by fever and systemic manifestations, including cutaneous, lung, ocular, and musculoskeletal issues,^2^ with thrombosis and myocardial infarctions in approximately 6% of patients.^3^ Nonischemic cardiac manifestations, such as pericarditis or myocarditis, have ocurred in 4% to 33% of cases, although no studies have focused on heart involvement in VEXAS syndrome.^1,2,3^

## Methods

We conducted a retrospective multicenter study in France from November 1, 2020, to June 30, 2024, to describe the clinical presentation, laboratory findings, imaging characteristics, treatments, and outcomes of nonischemic cardiac manifestations in patients with VEXAS syndrome with confirmed pathogenic *UBA1* variations.^2^ Patients with myocardial infarction or angina were excluded. The study was conducted according to the Declaration of Helsinki and received approval from the Cochin Hospital institutional review board, which waived consent because the data were deidentified. This report follows the reporting guidelines for case series.

## Results

Of 299 patients with VEXAS syndrome, 8 (2.7%) exhibited cardiac manifestations. Five patients (1.7%) with nonischemic cardiac events were included for analysis (Table); the 3 remaining patients with ischemic cardiac manifestations were excluded. All 5 patients were male (median age, 65 years [IQR, 65-72 years] at the onset of symptoms). The types of *UBA1* variations identified included p.Met41Thr, p.Met41Leu, and c118-1G>C. The median interval between initial VEXAS symptoms and cardiac involvement was 2.3 years (IQR, 2.0-2.5 years).

**Table.** Characteristics of Patients

Patient No./age at VEXAS symptoms, y	*UBA1* vaiation	VEXAS manifestations	Time between VEXAS symptoms and cardiac manifestations	CV history	CV symptoms	Results	Diagnosis	Cardiac treatment	VEXAS syndrome management	Outcome		Death
										Cardiac	VEXAS syndrome	
1/70s	p.M41T	Fever, skin lesions, lung involvement, lymphadenopathies, splenomegaly, venous thrombosis, arthralgia	(1) 0 y (2) 2 y	High blood pressure Tobacco use; CAD	Chest pain, NYHA class 1 dyspnea	Laboratory results, (1) increased troponins, CRP (2) increased troponins, NT-proBNP, CRP; imaging results, (1) TTE: pericardial effusion (2) TTE: LVEF 45% and hypokinesia; MRI: myocarditis; ECG results, (1) normal (2) negative T waves	(1) Pericarditis (2) Myocarditis	(1) Aspirin and β-blockers (2) Added loop diuretic	Anakinra, 100 mg/d	Regression	Improvement	Yes; not CV-related
2/70s	p.M41L	Skin lesions, chondritis, arthralgia, ocular involvement	2 y	High blood pressure; tobacco use	Heart failure	Laboratory results, increased CRP; imaging results, TTE: LVEF 35%; ECG results, tachycardia; atrial flutter	Heart failure with mildly reduced EF and arrythmia	HF treatment; antiarrhythmic agents; resynchronization; anticoagulation	Tocilizumab 162 mg/wk and corticosteroids	Worsening	Stable	Yes
3/60s	p.M41L	Skin lesions, venous thrombosis, lung involvement, ocular involvement, arthralgia, chondritis	6 y	None	Heart failure	Laboratory results, increased troponins, NT-proBNP, CRP; imaging results, CMR: vasculitis with multiple microinfarcts, necrosis sequelae, preserved LVEF; ECG results, tachycardia	Myocarditis	β-Blockers; loop diuretic; anticoagulation	Intravenous methylprednisolone 500 mg, 3 doses; infliximab	Worsening	Worsening	Yes; not CV-related
4/60s	c.118-1G>C	Fever, skin lesions, chondritis, lung involvement, myelodysplastic syndrome	2 y	Tobacco	Heart failure and arrythmia	Laboratory results, increased NT-proBNP, CRP; imaging results, TTE: pulmonary hypertension, pericardial effusion, mitral insufficiency; ECG results, complete AVB	3rd degree AVB and then APO	Loop diuretic pacemaker	Intravenous nethylprednisolone 1 g, 3 doses	Regression after pacemaker	Worsening	Yes; not CV-related
5/50s	p.M41T	Fever, skin lesions, chondritis, lymphadenopathies, kidney amyloid A amyloidosis	2.5 y	CAD and valve disease	None	Laboratory results, increased troponins, NT-proBNP, SAA, CRP; imaging results, TTE: aortic stenosis, LVEF 55%; CMR: cardiac amyloidosis; ECG results, tachycardia, microvoltage, negative T waves	Amyloid A amyloidosis	ACE inhibitors	Anakinra 100 mg 3 times per wk during dialysis session	Stable	Stable	No

Abbreviations: ACE, angiotensin-converting enzyme; APO, acute pulmonary edema; AVB, atrioventricular block; CAD, coronary artery disease; CMR, cardiac magnetic resonance imaging; CRP, C-reactive protein; CV, cardiovascular; ECG, electrocardiography; EF, ejection fraction; HF, heart failure; LVEF, left ventricular ejection fraction; MRI, magnetic resonance imaging; NT-proBNP, N-terminal pro-brain natriuretic peptide; NYHA, New York Heart Association; SAA, serum amyloid A; TTE, transthoracic echocardiography; VEXAS, vacuoles, E1 enzyme, X-linked, autoinflammatory, somatic.

Patient 1 experienced 2 cardiac events: pericarditis treated with aspirin and later myocarditis treated successfully with interleukin 1 inhibitor anakinra. Both cardiac manifestations occurred during a disease flare-up, including fever and skin lesions. He later died from an infection.

Patient 2 exhibited symptoms of acute heart failure and arrhythmia associated with clinical manifestations of VEXAS syndrome (eg, skin lesions, chondritis), which were managed with heart failure treatment and antiarrhythmic agents, alongside interleukin 6 inhibitor tocilizumab and corticosteroids; other VEXAS symptoms were stabilized. He died from cardiac arrest during anesthesia induction for kidney cancer surgery.

Patient 3 presented with acute left ventricular failure, myocarditis, and multiple myocardial infarctions (Figure); he also displayed multiple manifestations of VEXAS syndrome (eg, lung involvement, skin lesions, chondritis). He did not respond to treatment and died of intestinal ischemia, peritonitis, and bleeding.

**Figure.**
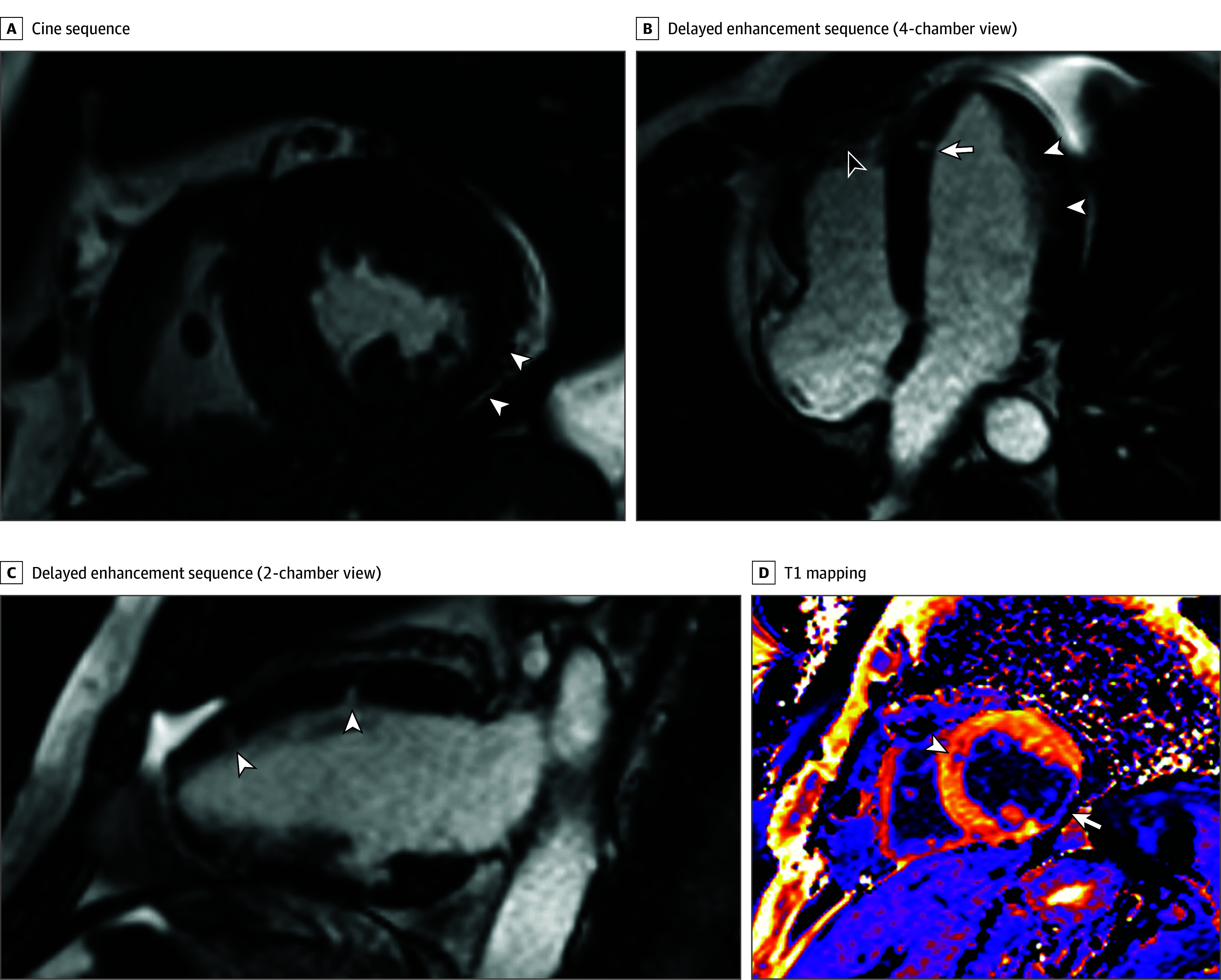
Cardiac Magnetic Resonance Imaging of Patient 3 Showing Myocardial Infarctions and Necrosis Sequalae A, Cine sequence, with midventricular short-axis view at end systole showing severe thinning of the inferolateral wall of the left ventricle (white arrows). B, Delayed enhancement sequence performed 10 minutes after gadolinium injection (4-chamber view), with subendocardial enhancement of the left ventricular wall (white arrowheads), nodular enhancement of the apical septum (white arrow), and enhancement of the right ventricular apex (black arrowhead). C, Delayed enhancement sequence performed 10 minutes after gadolinium injection (2-chamber view), with nodular foci of enhancement of the anterior wall of the left ventricle (arrowheads). D, T1 mapping performed 12 minutes after gadolinium injection, with midventricular short-axis view showing decreased signal of the inferolateral wall of the left ventricle (arrow) due to gadolinium retention and nodular focus of decreased signal in the anterior septum (arrowhead).

Patient 4 experienced acute heart failure, pulmonary hypertension, and third-degree atrioventricular block concomitant with a disease flare-up (eg, fever, chondritis), which was managed with pacemaker implantation and methylprednisolone. He died from acute respiratory failure.

Patient 5 exhibited cardiac inflammatory amyloidosis. Amyloid A amyloidosis was confirmed on kidney biopsy, and a cardiac magnetic resonance imaging scan was consistent with cardiac amyloid A amyloidosis. The patient was treated with anakinra, and both cardiac and VEXAS syndrome symptoms stabilized.^4^ He is still alive and receives dialysis 3 times per week.

## Discussion

Nonischemic cardiac involvement in VEXAS syndrome is rare, with inflammatory diseases such as pericarditis, myocarditis, and heart vasculopathy being the primary diagnoses. The study suggests that these cardiac manifestations are directly associated with the systemic inflammation, which is associated with VEXAS syndrome. The successful treatment of myocarditis with anakinra in 1 case further supports this connection. However, the exact mechanisms remain unclear, with hypotheses suggesting myeloid cell infiltration carrying an *UBA1* variation or small-vessel vasculopathy as causes.^5,6^

The retrospective design prevented a standardized assessment of cardiac involvement in all patients. By excluding patients with myocardial infarction and angina, we may have excluded some cases of coronary artery vasculitis directly associated with VEXAS syndrome.

Nonischemic cardiac manifestations are rare in VEXAS syndrome but do occur during disease flare-ups, in addition to myocardial infarctions that are more frequent.^3^ Controlling inflammation in VEXAS syndrome is crucial to prevent cardiac involvement and avoid complications such as inflammatory amyloidosis. The high mortality rate in this series underscores the disease’s severity. Further research, including expanded magnetic resonance imaging and cardiac biopsies, is needed to better understand the mechanisms of cardiac involvement in VEXAS syndrome.

## Notes

Supplement 1. **Nonauthor Collaborators.** FRENVEX Group

Supplement 2.
Data Sharing Statement
