# Supplementary material for: Nonischemic Cardiac Manifestations in VEXAS Syndrome
Source: JAMA Netw Open. 2024 Dec 12;7(12):e2450251. doi: 10.1001/jamanetworkopen.2024.50251 (PMC11638797; doi:10.1001/jamanetworkopen.2024.50251)
Supplement: Supplement 1. — Nonauthor Collaborators. FRENVEX Group [file jamanetwopen-e2450251-s001.pdf]

Supplemental Online Content: Nonauthor Collaborators
*First name, last name, and suffix (if applicable) are required and will appear in PubMed.

***Group Name(s): The FRENVEX Group**

*First Name and Middle Initial(s)	*Last Name	*Suffix (eg, Jr, III)	Academic Degrees	Institution	Location (city, state/province, country)	Role or Contribution, eg, chair, principal investigator	Group (if more than 1 Group listed in the byline) and/or Subgroup (eg, Steering Committee)
Jérome	HADJADJ	NA	MD-PhD	Assistance Publique - Hôpitaux Paris	Paris, France	Collaborators	FRENVEX
Yann	NGUYEN	NA	MD-PhD	Assistance Publique - Hôpitaux Paris	Clichy, France	Collaborators	FRENVEX
Dalila	MOULOUDJ	NA	MD	Assistance Publique - Hôpitaux Paris	Paris, France	Collaborators	FRENVEX
Mael	HEIBLIG	NA	MD-PhD	Hospices Civils de Lyon	Lyon, France	Collaborators	FRENVEX
Hassina	ALOUI	NA	MD	Assistance Publique - Hôpitaux Paris	Paris, France	Collaborators	FRENVEX
Chloé	MC AVOY	NA	MD	Assistance Publique - Hôpitaux Paris	Paris, France	Collaborators	FRENVEX
Samuel	ARDOIS	NA	MD	CHU Rennes	Rennes, France	Collaborators	FRENVEX
Corrado	CAMPOCHIARO	NA	MD	IRCCS San Raffaele Hospital	Milan, Italie	Collaborators	FRENVEX
Alexandre	MARIA	NA	MD	St Eloi Hospital	Montpellier, France	Collaborators	FRENVEX
Cyrille	COUSTAL	NA	MD	St Eloi Hospital	Montpellier, France	Collaborators	FRENVEX
Thibault	COMONT	NA	MD	CHU Toulouse	Toulouse, France	Collaborators	FRENVEX
Estibaliz	LAZARO	NA	MD	CHU Bordeaux	Bordeaux, France	Collaborators	FRENVEX
François	LIFERMANN	NA	MD	Centre Hospitalier Dax	Dax, France	Collaborators	FRENVEX
Guillaume	LE GUENNO	NA	MD	CHU Estaing	Clermont-Ferrand, France	Collaborators	FRENVEX
Vincent	GROBOST	NA	MD	CHU Estaing	Clermont-Ferrand, France	Collaborators	FRENVEX
Roderau	OUTH	NA	MD	Perpignan University	Perpignan, France	Collaborators	FRENVEX
Julien	CAMPAGNE	NA	MD	Hopital Robert Schuman	Metz, France	Collaborators	FRENVEX
Anais	DOR-ETIENNE	NA	MD	Hopital Robert Schuman	Metz, France	Collaborators	FRENVEX
Alice	GARNIER	NA	MD	CHU Nantes	Nantes, France	Collaborators	FRENVEX
Yvan	JAMILLOUX	NA	MD-PhD	Hospices Civils de Lyon	Lyon, France	Collaborators	FRENVEX
Antoine	DOSSIER	NA	MD	Assistance Publique - Hôpitaux Paris	Paris, France	Collaborators	FRENVEX
Maxime	SAMSON	NA	MD-PhD	CHU Dijon-Bourgogne	Dijon, France	Collaborators	FRENVEX

*First name, last name, and suffix (if applicable) are required and will appear in PubMed.

*First Name and Middle Initial(s)	*Last Name	*Suffix (eg, Jr, III)	Academic Degrees	Institution	Location (city, state/province, country)	Role or Contribution, eg, chair, principal investigator	Group (if more than 1 Group listed in the byline) and/or Subgroup (eg, Steering Committee)
Sylvain	AUDIA	NA	MD	CHU Dijon-Bourgogne	Dijon, France	Collaborators	FRENVEX
Barbara	NICOLAS	NA	MD	CHU Dijon-Bourgogne	Dijon, France	Collaborators	FRENVEX
Baptiste	DE MALEPRADE	NA	MD	CHU Rouen	Rouen, France	Collaborators	FRENVEX
Benjamin	DE SAINTE MARI	NA	MD	CHU de la Timone	Marseille, France	Collaborators	FRENVEX
Benoit	FAUCHER	NA	MD	CHU de la Timone	Marseille, France	Collaborators	FRENVEX
Jean-David	BOUAZIZ	NA	MD	Assistance Publique - Hôpitaux Paris	Paris, France	Collaborators	FRENVEX
Jonathan	BRONER	NA	MD	CHU Nimes	Nimes, France	Collaborators	FRENVEX
Cyril	DUMAIN	NA	MD	CHU Nimes	Nimes, France	Collaborators	FRENVEX
Carole	ANTOINE	NA	MD	Hôpital Militaire Sainte-Anne	Toulon, France	Collaborators	FRENVEX
Benjamin	CARPENTIER	NA	MD	Hôpital St Vincent de Paul	Lille, France	Collaborators	FRENVEX
Brice	CASTEL	NA	MD	CH de Lourdes	Lourdes, France	Collaborators	FRENVEX
Céline	LARTIGAU-ROUS	NA	MD	CH Ouest Réunion	Saint Paul, France	Collaborators	FRENVEX
Etienne	CRICKX	NA	MD-PhD	Assistance Publique - Hôpitaux Paris	Paris, France	Collaborators	FRENVEX
Geoffroy	VOLLE	NA	MD	Assistance Publique - Hôpitaux Paris	Paris, France	Collaborators	FRENVEX
Damien	FAYARD	NA	MD	CHU Gabriel Montpied	Clermont-Ferrand, France	Collaborators	FRENVEX
Paul	DECKER	NA	MD	CHU de Nancy	Nancy, France	Collaborators	FRENVEX
Thomas	MOULINET	NA	MD	CHU de Nancy	Nancy, France	Collaborators	FRENVEX
Anael	DUMONT	NA	MD	CHU de Caen	Caen, France	Collaborators	FRENVEX
Alexandre	NGUYEN	NA	MD	CHU de Caen	Caen, France	Collaborators	FRENVEX
Achille	AOUBA	NA	MD	CHU de Caen	Caen, France	Collaborators	FRENVEX
Jean-Philippe	MARTELLOSIO	NA	MD	CHU de Poitiers	Poitiers, France	Collaborators	FRENVEX
Matthieu	LEVAVASSEUR	NA	MD	CH Genevois	Annecy, France	Collaborators	FRENVEX
Sébastien	PUIGRENIER	NA	MD	CH de Boulogne-Sur-Mer	Boulogne-Sur-Mer, France	Collaborators	FRENVEX
Pascale	ANTOINE	NA	MD	CH de Boulogne-Sur-Mer	Boulogne-Sur-Mer, France	Collaborators	FRENVEX
Jean-Thomas	GIRAUD	NA	MD	Tarbes Hospital	Tarbes, France	Collaborators	FRENVEX
Olivier	HERMINE	NA	MD-PhD	Assistance Publique - Hôpitaux Paris	Paris, France	Collaborators	FRENVEX

Supplemental Online Content: Nonauthor Collaborators
*First name, last name, and suffix (if applicable) are required and will appear in PubMed.

*First Name and Middle Initial(s)	*Last Name	*Suffix (eg, Jr, III)	Academic Degrees	Institution	Location (city, state/province, country)	Role or Contribution, eg, chair, principal investigator	Group (if more than 1 Group listed in the byline) and/or Subgroup (eg, Steering Committee)
Carole	LACOUT	NA	MD	CHU Angers	Angers, France	Collaborators	FRENVEX
Nihal	MARTIS	NA	MD	Hôpital Archet 1	Nice, France	Collaborators	FRENVEX
Jean-Denis	KARAM	NA	MD	CHU Amiens	Amiens, France	Collaborators	FRENVEX
François	CHASSET	NA	MD-PhD	Assistance Publique - Hôpitaux Paris	Paris, France	Collaborators	FRENVEX
Laurent	ARNAUD	NA	MD-PhD	Hôpitaux Universitaires de Strasbour	Strasbourg, France	Collaborators	FRENVEX
Paola	MARIANETTI GU	NA	MD	Reims Champagne-Ardenne Universi	Reims, France	Collaborators	FRENVEX
Christophe	DELIGNY	NA	MD	CHU de Martinique	Fort-De-France, Martiniqu	Collaborators	FRENVEX
Thibaud	CHAZAL	NA	MD	The Fondation Aldophe de Rothschild Hospital	Paris, France	Collaborators	FRENVEX
Pascal	WOAYE-HUNE	NA	MD	CHD Vendée	La Roche-Sur-Yon, France	Collaborators	FRENVEX
Marielle	ROUX-SAUVAT	NA	MD	Hôpital Pierre Oudaut	Bourgoin-Jallieu, France	Collaborators	FRENVEX
Aurore	MEYER	NA	MD	Hôpitaux Universitaires de Strasbour	Strasbourg, France	Collaborators	FRENVEX
Pierre	HIRSCH	NA	MD	Assistance Publique - Hôpitaux Paris	Paris, France	Collaborators	FRENVEX
Noémie	ABISROR	NA	MD	Assistance Publique - Hôpitaux Paris	Paris, France	Collaborators	FRENVEX
Pierre	FENAUX	NA	MD	Assistance Publique - Hôpitaux Paris	Paris, France	Collaborators	FRENVEX
Olivier	KOSMIDER	NA	MD-PhD	Assistance Publique - Hôpitaux Paris	Paris, France	Collaborators	FRENVEX
Vincent	JACHIER	NA	MD	Assistance Publique - Hôpitaux Paris	Paris, France	Collaborators	FRENVEX
Olivier	FAIN	NA	MD-PhD	Assistance Publique - Hôpitaux Paris	Paris, France	Collaborators	FRENVEX
