# Supplementary material for: Nonischemic Cardiac Manifestations in VEXAS Syndrome
Source: JAMA Netw Open. 2024 Dec 12;7(12):e2450251. doi: 10.1001/jamanetworkopen.2024.50251 (PMC11638797; doi:10.1001/jamanetworkopen.2024.50251)
Supplement: Supplement 2. — Data Sharing Statement [file jamanetwopen-e2450251-s002.pdf]

Data Sharing Statement

Robert. Nonischemic Cardiac Manifestations in VEXAS Syndrome. *JAMA Netw Open*. Published December 12, 2024. doi:10.1001/jamanetworkopen.2024.50251

Data
Data available: No
